# Assessment and Retrieval of Aspirated Tracheoesophageal Prosthesis in the Ambulatory Setting

**DOI:** 10.1155/2018/9369602

**Published:** 2018-09-13

**Authors:** Karuna Dewan, Andrew Erman, Jennifer L. Long, Dinesh K. Chhetri

**Affiliations:** Department of Head and Neck Surgery, David Geffen School of Medicine at UCLA, Los Angeles, CA 90095, USA

## Abstract

Tracheoesophageal prosthesis (TEP) is the most common voice restoration method following total laryngectomy. Prosthesis extrusion and aspiration occurs in 3.9% to 6.7% and causes dyspnea. Emergency centers are unfamiliar with management of the aspirated TEP. Prior studies report removal of aspirated TEP prostheses under general anesthesia. Laryngectomees commonly have poor pulmonary function, posing increased risks for complications of general anesthesia. We present a straightforward approach to three cases of aspirated TEP prosthesis removed in the ambulatory setting. In each case, aspirated TEP was diagnosed with flexible bronchoscopy under local anesthesia at the time of consultation, and all prostheses were retrieved atraumatically using a biopsy grasper forceps inserted via the side channel of the bronchoscope. The aspirated TEP prosthesis can be safely and efficiently removed via bedside bronchoscopy.

## 1. Introduction

Tracheoesophageal puncture with voice prosthesis placement is the gold standard for voice restoration following total laryngectomy [1]. It provides the most natural voice and is easiest to use compared to other rehabilitation methods [2, 3]. Leakage and malfunction are the most common complications; however, the prosthesis also often becomes dislodged [4]. The dislodged prosthesis may be aspirated, with reported incidence of 3.9% to 6.7% [5, 6]. Aspiration of the voice prosthesis into the trachea may cause significant airway obstruction. Removing aspirated TEPs has traditionally required emergent rigid bronchoscopy under general anesthesia in the operating room. Rao Kadam et al. described the difficult retrieval of an aspirated speaking valve using rigid bronchoscopy under general anesthesia. In a patient with a stoma, difficulties include ventilation issues as well as mechanical issues with the introduction of a rigid bronchoscope into the airway through the neck rather than through the mouth as it was designed to be used [7].

The three cases presented here demonstrate management of an aspirated TEP in the ambulatory setting with flexible bronchoscopy and local anesthetics, avoiding general anesthesia and the associated risk factors and costs. Despite being straightforward, this practice is unfamiliar to community otolaryngologists. Two of the presented patients were previously seen at outside institutions and transferred for a higher care level. The UCLA Institutional Review Board exempted this study.

## 2. Case 1

A 67-year-old male underwent total laryngectomy and secondary tracheoesophageal prosthesis placement 15 years prior. He was proficient at the use and care of his TEP. He lost his TEP and presented to his speech language pathologist for replacement. He did not recall how or when the TEP was dislodged; thus, it was unclear if he swallowed or aspirated it. After developing mild dyspnea, an emergency room chest X-ray suggested a foreign body. The patient was subsequently transferred to our head and neck surgery office. He reported increased mucus production, coughing, fullness in his chest, and inability to fully catch his breath.

**Figure 1:**
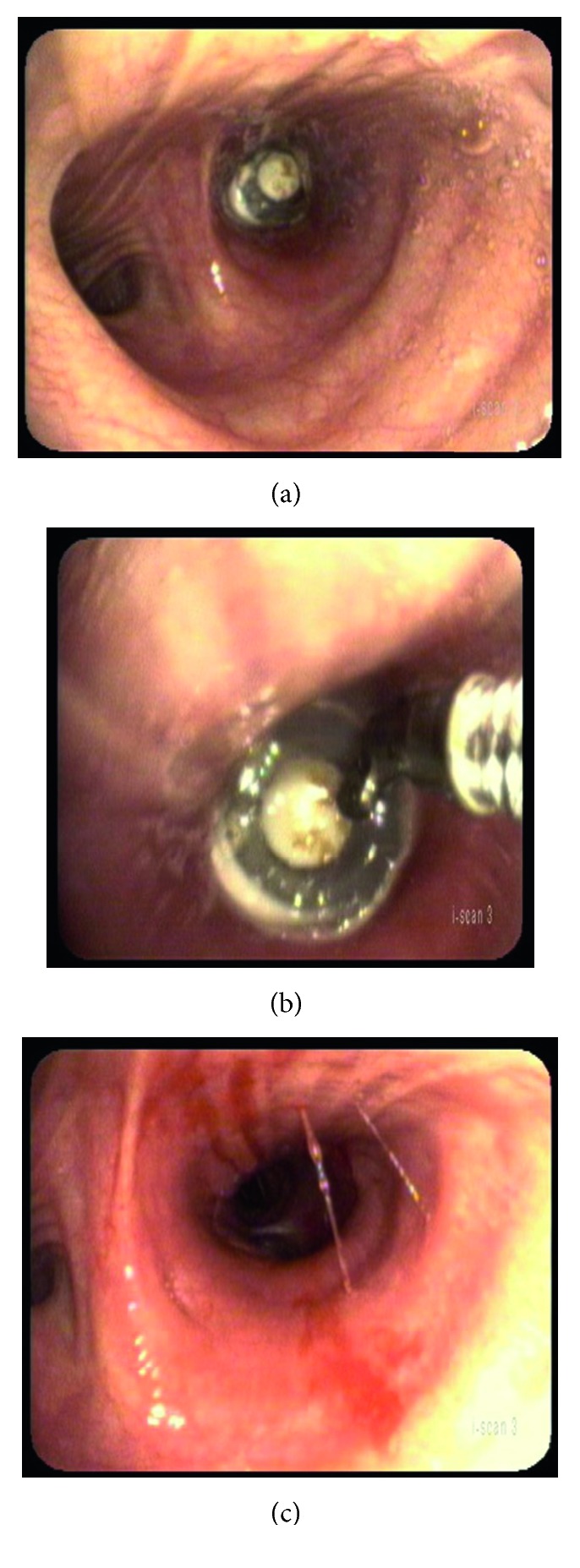
(a) Aspirated prosthesis in the left mainstem bronchus. It is situated such that the one-way valve allowed only inspiration. (b) Flexible biopsy forceps grabbing the prosthesis. (c) Left mainstem bronchus after atraumatic prosthesis removal.

To examine the airway for possible aspirated TEP, topical 4% lidocaine was sprayed into the stoma. With the patient sitting upright in the examination chair, flexible bronchoscopy was performed using a transnasal esophagoscope (KayPentax EE-1580). The prosthesis was found in the left mainstem bronchus (Figure 1).

The TEP's one-way valve orientation allowed air inhalation but not exhalation, trapping air in the lung. The prosthesis was retrieved without difficulty by grasping it with biopsy forceps (Olympus Endobronchial Alligator Jaw Forceps, FB 15C-1) passed through the esophagoscope side channel, then withdrawing the entire bronchoscope out of the trachea-stoma. After removal, the airway was reexamined demonstrating no additional foreign body or injury. The patient tolerated the procedure without discomfort and felt immediate relief of dyspnea. Postremoval chest X-ray failed to show any abnormality.

## 3. Case 2

**Figure 2:**
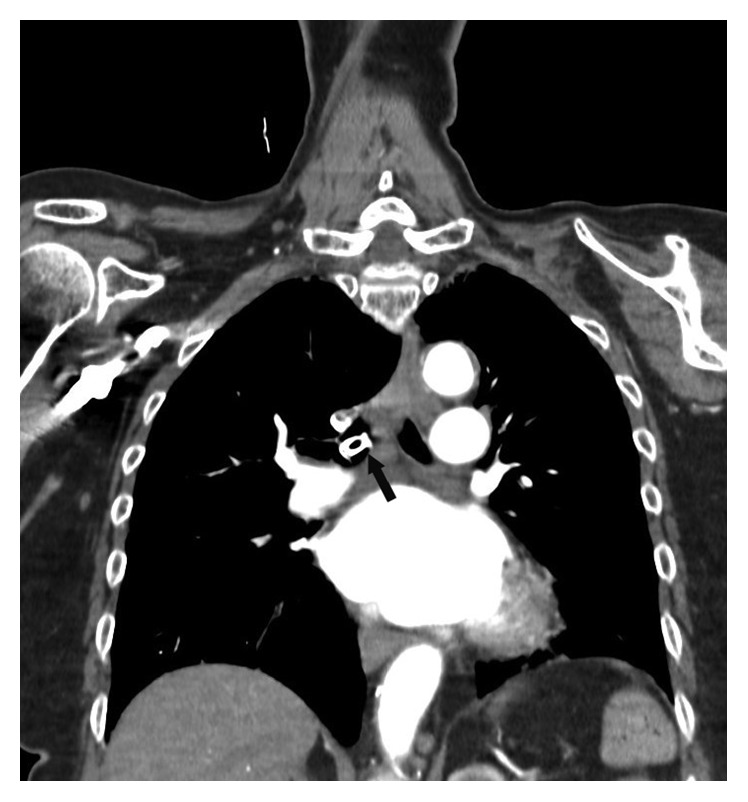
Coronal CT scan demonstrating aspirated TEP in the right mainstem bronchus.

A 72-year-old female 20 years following laryngectomy, free flap reconstruction, and radiation had been using a TEP successfully. While cleaning her prosthesis, it dislodged and was aspirated. She initially presented to an outside hospital acutely short of breath, requiring supplemental oxygen to maintain saturations >90%. CT chest demonstrated a radiopaque foreign body in the right mainstem bronchus (Figure 2).

**Figure 3:**
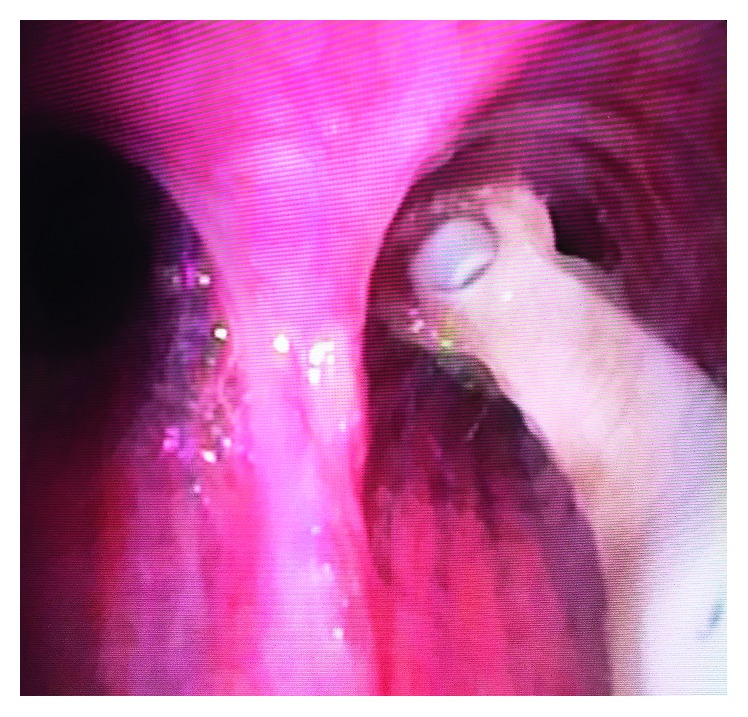
Aspirated TEP from the right mainstem bronchus.

She was transferred to the emergency department (ED) at our institution as the local consultants recommended “higher level of care.” The otolaryngology service evaluated the patient in the ED and performed bedside flexible bronchoscopy (Olympus BF-H190) after topical lidocaine spray. The aspirated prosthesis was found in the right mainstem bronchus (Figure 3).

It was removed atraumatically by grasping with the flexible biopsy forceps then withdrawing the entire bronchoscope. There were no other injuries or remaining foreign bodies. She felt immediate relief. A red rubber catheter was placed through her tracheoesophageal puncture to stent the tract. She was discharged home with next day follow-up with her speech-language pathologist (SLP) for prosthesis replacement.

## 4. Case 3

**Figure 4:**
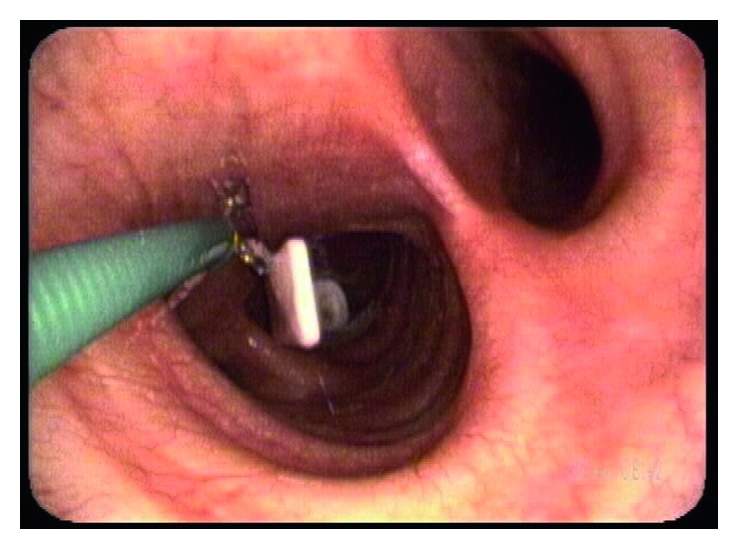
Removal of aspirated TEP from the right mainstem bronchus.

A 56-year-old male, 8 years postlaryngectomy with free flap reconstruction, proficient at changing his own prosthesis, presented to our clinic with 2 days of worsening dyspnea. The patient had not noticed aspirating his prosthesis after changing it. He underwent flexible bronchoscopy under topical anesthesia while sitting upright. A TEP lodged in the right mainstem bronchus was removed with biopsy forceps via the endoscope channel (Figure 4). His dyspnea immediately resolved.

## 5. Discussion

A “lost” tracheoesophageal prosthesis may be swallowed, aspirated, or expelled. Patients with aspirated TEP present with a range of complaints from intermittent cough to more significant airway distress. The prosthesis has a one-way valve, which may compound the airway problem and can potentially lead to pulmonary collapse or overinflation.

All patients who lose their voice prosthesis should undergo a chest X-ray to evaluate for aspirated foreign body. Some prostheses have a radiopaque ring for easy identification. Occasionally, the aspirated TEP may not be reliably demonstrated on imaging, leading to delayed diagnosis. Continued vigilance for airway symptoms must be undertaken by both patient and physician. All TEP patients should be counseled to present in a timely manner to the otolaryngology office in case of lost prosthesis, so that office tracheobronchoscopy can be performed with topical anesthesia and any flexible endoscope. If a prosthesis is found in the airway, it can be removed using flexible biopsy forceps that can be placed through the scope channel or just adjacent to the scope. The greatest advantage to this method of TEP recovery is the immediate relief of symptoms. While documented cases of aspirated TEP removal using this method are rare, Chorney et al. report the removal of an aspirated screw in this manner. The patient presented with dysphagia, chest pain, cough, and wheeze. An esophogram demonstrated the presence of a radiopaque foreign body in the right lower lung. The thoracic surgery team was able to remove this with flexible bronchoscopy leading to immediate relief of symptoms [8]. Therefore, the method described in this paper has previously been useful for removal of other airway foreign bodies in the laryngectomy patient.

Failure to present in a timely manner for management of the lost TEP can also lead to closure of the tracheoesophageal puncture tract. This is the most common reason for emergency room presentation after prosthesis loss. Most patients following aspiration of the speech prosthesis are asymptomatic. However, this does not exclude the danger of an aspirated foreign body. Grosu et al. reported the removal of an aspirated speech prothesis, embedded in the carina, using bronchoscopy under general anesthesia. That particular patient was asymptomatic, presenting to the ED after the foreign body was noted on CT scan of the chest [9]. While the patient did not demonstrate any cough, shortness of breath, or hemoptysis, the prolonged presence of the foreign body in the airway places that patient at risk for the development of complications including pneumonia and erosion through the tracheal wall. Therefore, upon loss of a prosthesis, it is important to verify that it is not in the airway, even if a patient is asymptomatic.

## 6. Conclusions

Tracheoesophageal prosthesis aspiration can lead to significant morbidity. Every case of lost TEP must be assessed minimally with a chest X-ray and ideally also by an otolaryngologist. This report demonstrates the ease and applicability of awake flexible endoscopic removal of aspirated TEP under topical anesthesia. This straightforward procedure can be performed by any otolaryngologist, avoids general anesthesia, and immediately resolves symptoms.
